# Supplementary material for: A comparative study of the performance of ten metaheuristic algorithms for parameter estimation of solar photovoltaic models
Source: PeerJ Comput Sci. 2025 Jan 27;11:e2646. doi: 10.7717/peerj-cs.2646 (PMC11784897; doi:10.7717/peerj-cs.2646)
Supplement: Supplemental Information 1 [file peerj-cs-11-2646-s001.pdf]

Table 1: The ranking of algorithms COA(1), GOA, COA(2), CPO, and GO for the SDM.

		COA(1)			GOA			COA(2)			CPO			GO		
		ν_1	ν_2	ν_3	ν_1	ν_2	ν_3	ν_1	ν_2	ν_3	ν_1	ν_2	ν_3	ν_1	ν_2	ν_3
C_1	**Score**	4.60E-01	5.81E-01	1.82E-01	4.20E-01	5.41E-01	3.45E-01	3.40E-01	5.73E-01	1.72E-01	5.88E-01	6.13E-01	4.99E-02	5.07E-01	4.85E-01	1.48E-01
			4.21E-01			4.31E-01			3.56E-01			4.60E-01			4.12E-01	
	**Rank**		3			5			9			8			9	
C_2	**Score**	9.33E-01	4.87E-01	1.13E-01	6.76E-01	5.78E-01	1.28E-01	2.69E-01	5.74E-01	3.93E-01	4.86E-01	4.67E-01	4.62E-01	1.22E-01	4.11E-01	1.76E-01
			6.17E-01			5.15E-01			3.76E-01			4.75E-01			2.08E-01	
	**Rank**		11			11			12			10			3	
C_3	**Score**	9.33E-01	6.15E-01	1.88E-01	5.08E-01	5.08E-01	2.46E-01	2.52E-01	5.39E-01	1.85E-01	5.37E-01	4.81E-01	5.21E-01	5.75E-01	3.69E-01	2.63E-01
			6.67E-01			4.42E-01			3.07E-01			5.19E-01			4.46E-01	
	**Rank**		13			7			6			14			10	
C_4	**Score**	5.11E-01	5.68E-01	1.72E-01	5.38E-01	4.33E-01	2.40E-01	2.38E-01	5.97E-01	3.89E-01	2.81E-01	4.77E-01	2.69E-01	3.40E-02	4.59E-01	2.55E-01
			4.40E-01			4.37E-01			3.65E-01			3.27E-01			1.95E-01	
	**Rank**		4			6			11			3			1	
C_5	**Score**	9.67E-01	5.16E-01	2.90E-01	5.31E-01	3.85E-01	5.11E-01	3.22E-01	5.21E-01	5.61E-01	3.69E-01	4.96E-01	4.22E-01	2.82E-01	5.40E-01	2.77E-01
			6.85E-01			4.89E-01			4.31E-01			4.14E-01			3.45E-01	
	**Rank**		14			10			15			5			6	
C_6	**Score**	9.67E-01	5.92E-01	2.39E-01	3.33E-01	3.64E-01	4.03E-01	2.78E-01	5.29E-01	2.42E-01	4.99E-01	4.54E-01	5.06E-01	5.24E-01	4.42E-01	4.46E-01
			6.91E-01			3.58E-01			3.32E-01			4.89E-01			4.84E-01	
	**Rank**		15			1			8			12			14	
C_7	**Score**	2.81E-01	5.80E-01	4.71E-01	4.02E-01	4.70E-01	2.82E-01	3.11E-01	5.09E-01	4.24E-01	4.46E-02	5.49E-01	5.66E-01	6.35E-01	4.39E-01	4.28E-01
			4.03E-01			3.89E-01			3.89E-01			3.01E-01			5.34E-01	
	**Rank**		2			3			14			2			15	
C_8	**Score**	4.41E-01	5.52E-01	1.75E-01	6.82E-01	5.33E-01	3.81E-01	2.39E-01	5.58E-01	1.65E-01	5.16E-01	3.80E-01	3.59E-01	1.69E-01	6.15E-01	1.80E-01
			4.02E-01			5.69E-01			3.00E-01			4.42E-01			2.83E-01	
	**Rank**		1			14			5			6			5	
C_9	**Score**	8.69E-01	4.59E-01	1.37E-01	5.28E-01	5.67E-01	4.67E-01	2.29E-01	4.82E-01	1.60E-01	5.85E-01	5.24E-01	4.55E-01	3.87E-02	4.85E-01	2.69E-01
			5.84E-01			5.22E-01			2.75E-01			5.37E-01			2.08E-01	
	**Rank**		8			12			4			15			4	
C_{10}	**Score**	5.71E-01	4.45E-01	4.87E-01	3.55E-01	4.03E-01	4.10E-01	2.35E-01	5.80E-01	4.61E-01	4.74E-01	5.27E-01	3.52E-01	6.04E-01	5.31E-01	1.31E-01
			5.19E-01			3.81E-01			3.78E-01			4.57E-01			4.68E-01	
	**Rank**		6			2			13			7			13	
C_{11}	**Score**	7.67E-01	4.64E-01	1.43E-01	4.04E-01	6.87E-01	4.26E-01	2.84E-01	4.05E-01	4.54E-01	5.25E-01	5.84E-01	3.03E-01	4.37E-01	4.79E-01	5.00E-01
			5.35E-01			4.80E-01			3.57E-01			4.84E-01			4.63E-01	
	**Rank**		7			9			10			11			12	
C_{12}	**Score**	9.67E-01	3.65E-01	1.61E-01	4.63E-01	5.06E-01	2.21E-01	2.00E-01	3.14E-01	1.67E-01	5.69E-01	6.05E-01	1.34E-01	6.54E-02	4.72E-01	1.94E-01
			6.15E-01			4.13E-01			2.21E-01			4.69E-01			1.99E-01	
	**Rank**		9			4			1			9			2	
C_{13}	**Score**	6.06E-01	6.03E-01	1.27E-01	6.50E-01	5.31E-01	4.05E-01	2.19E-01	4.30E-01	2.05E-01	4.42E-02	4.91E-01	4.21E-01	4.03E-01	5.84E-01	2.34E-01
			4.86E-01			5.59E-01			2.68E-01			2.50E-01			4.06E-01	
	**Rank**		5			13			2			1			8	
C_{14}	**Score**	9.67E-01	4.12E-01	1.17E-01	5.44E-01	4.98E-01	2.73E-01	1.92E-01	4.85E-01	2.10E-01	4.99E-01	5.15E-01	5.56E-01	2.67E-01	5.34E-01	4.38E-01
			6.15E-01			4.65E-01			2.70E-01			5.17E-01			3.77E-01	
	**Rank**		10			8			3			13			7	
C_{15}	**Score**	9.33E-01	4.91E-01	2.84E-01	5.84E-01	5.71E-01	5.61E-01	2.32E-01	5.19E-01	2.68E-01	3.73E-01	3.59E-01	3.98E-01	4.07E-01	5.75E-01	4.25E-01
			6.60E-01			5.75E-01			3.13E-01			3.76E-01			4.54E-01	
	**Rank**		12			15			7			4			11	

Table 2: The ranking of algorithms APO, MOA, SBOA, EOA, and TVETBO for the SDM.

		**APO**			**MOA**			**SBOA**			**EOA**			**TVETBO**		
		ν_1	ν_2	ν_3	ν_1	ν_2	ν_3	ν_1	ν_2	ν_3	ν_1	ν_2	ν_3	ν_1	ν_2	ν_3
C_1	**Score**	6.71E-01	5.03E-01	2.24E-01	5.36E-01	4.73E-01	2.12E-01	6.06E-01	4.55E-01	1.64E-01	2.57E-01	4.66E-01	2.54E-01	6.20E-01	4.64E-01	1.12E-01
			5.17E-01			4.39E-01			4.58E-01			3.09E-01			4.54E-01	
	**Rank**		13			2			4			11			2	
C_2	**Score**	5.13E-01	4.48E-01	3.64E-01	9.67E-01	4.56E-01	2.79E-01	4.29E-01	5.31E-01	4.68E-01	1.10E-01	4.87E-01	4.56E-01	6.96E-01	4.97E-01	4.45E-01
			4.60E-01			6.67E-01			4.64E-01			2.91E-01			5.83E-01	
	**Rank**		11			10			5			8			13	
C_3	**Score**	2.84E-01	5.55E-01	1.46E-01	4.63E-01	4.60E-01	2.83E-01	9.67E-01	6.33E-01	4.96E-01	7.20E-02	4.05E-01	2.17E-01	7.29E-01	4.90E-01	5.26E-02
			3.18E-01			4.17E-01			7.66E-01			1.92E-01			5.00E-01	
	**Rank**		5			1			15			1			5	
C_4	**Score**	5.74E-01	3.95E-01	2.42E-01	4.92E-01	5.71E-01	5.44E-01	6.90E-01	3.86E-01	5.96E-01	1.53E-01	3.59E-01	4.78E-01	6.77E-01	6.38E-01	3.22E-01
			4.46E-01			5.25E-01			5.91E-01			2.86E-01			5.78E-01	
	**Rank**		9			3			11			7			12	
C_5	**Score**	4.55E-01	6.01E-01	1.20E-01	5.50E-01	7.02E-01	3.09E-01	5.96E-01	4.62E-01	6.21E-01	1.51E-01	5.04E-01	4.23E-01	6.31E-01	5.40E-01	6.63E-01
			4.08E-01			5.28E-01			5.68E-01			3.07E-01			6.16E-01	
	**Rank**		6			5			10			10			15	
C_6	**Score**	2.12E-01	4.57E-01	2.71E-01	9.67E-01	8.12E-01	5.96E-01	5.22E-01	5.02E-01	1.81E-01	5.09E-01	4.86E-01	4.47E-01	5.91E-01	5.87E-01	3.34E-01
			2.88E-01			8.35E-01			4.32E-01			4.88E-01			5.26E-01	
	**Rank**		4			15			3			12			8	
C_7	**Score**	6.12E-01	4.66E-01	1.45E-01	5.39E-01	4.93E-01	5.39E-01	4.08E-01	4.72E-01	5.85E-01	1.33E-01	4.14E-01	3.33E-01	6.31E-01	4.76E-01	3.97E-01
			4.59E-01			5.27E-01			4.68E-01			2.53E-01			5.34E-01	
	**Rank**		10			4			6			4			11	
C_8	**Score**	5.17E-01	4.51E-01	1.92E-01	5.83E-01	7.01E-01	4.06E-01	9.33E-01	5.82E-01	3.36E-01	1.18E-01	5.98E-01	3.89E-01	6.43E-01	4.14E-01	4.21E-01
			4.19E-01			5.68E-01			6.96E-01			3.06E-01			5.30E-01	
	**Rank**		8			7			13			9			9	
C_9	**Score**	1.27E-01	4.20E-01	1.02E-01	5.56E-01	7.35E-01	6.24E-01	5.78E-01	4.83E-01	5.46E-01	6.79E-01	5.73E-01	1.97E-01	6.05E-01	4.38E-01	2.76E-01
			1.94E-01			6.18E-01			5.46E-01			5.32E-01			4.81E-01	
	**Rank**		1			8			9			14			4	
C_{10}	**Score**	5.79E-01	4.26E-01	3.84E-01	9.67E-01	4.92E-01	5.08E-01	5.84E-01	9.01E-01	8.17E-01	1.22E-01	4.68E-01	2.35E-01	5.20E-01	4.48E-01	2.94E-01
			4.92E-01			7.33E-01			7.21E-01			2.37E-01			4.46E-01	
	**Rank**		12			14			14			3			1	
C_{11}	**Score**	4.17E-01	5.31E-01	3.09E-01	4.88E-01	6.37E-01	6.20E-01	4.66E-01	4.89E-01	5.37E-01	5.03E-02	4.61E-01	2.98E-01	5.97E-01	4.67E-01	4.35E-01
			4.18E-01			5.58E-01			4.89E-01			2.15E-01			5.24E-01	
	**Rank**		7			6			7			2			7	
C_{12}	**Score**	1.51E-01	3.98E-01	1.11E-01	9.18E-01	6.55E-01	4.22E-01	6.82E-01	5.29E-01	5.20E-01	6.69E-01	4.57E-01	2.12E-01	4.95E-01	5.67E-01	3.49E-01
			2.03E-01			7.28E-01			6.03E-01			5.02E-01			4.76E-01	
	**Rank**		2			13			12			13			3	
C_{13}	**Score**	7.10E-01	5.66E-01	2.01E-01	9.67E-01	4.66E-01	4.72E-01	5.79E-01	5.17E-01	5.02E-01	8.98E-02	4.36E-01	4.36E-01	7.55E-01	3.98E-01	5.18E-01
			5.47E-01			7.18E-01			5.44E-01			2.63E-01			6.07E-01	
	**Rank**		15			12			8			6			14	
C_{14}	**Score**	5.44E-01	5.11E-01	5.68E-01	9.00E-01	5.18E-01	5.50E-01	3.51E-01	4.33E-01	4.39E-01	5.10E-02	4.19E-01	5.01E-01	5.95E-01	4.96E-01	4.42E-01
			5.42E-01			7.17E-01			3.93E-01			2.55E-01			5.32E-01	
	**Rank**		14			11			2			5			10	
C_{15}	**Score**	1.16E-01	5.19E-01	3.83E-01	8.67E-01	5.05E-01	3.93E-01	4.88E-01	5.02E-01	9.49E-02	7.51E-01	5.04E-01	3.82E-01	5.99E-01	3.00E-01	5.88E-01
			2.83E-01			6.58E-01			3.93E-01			5.97E-01			5.21E-01	
	**Rank**		3			9			1			15			6	

Table 3: The ranking of algorithms COA(1), GOA, COA(2), CPO, and GO for the DDM.

		**COA(1)**			**GOA**			**COA(2)**			**CPO**			**GO**		
		ν_1	ν_2	ν_3	ν_1	ν_2	ν_3	ν_1	ν_2	ν_3	ν_1	ν_2	ν_3	ν_1	ν_2	ν_3
C_1	**Score**	5.96E-01	5.81E-01	1.82E-01	4.20E-01	4.83E-01	3.45E-01	3.40E-01	5.44E-01	1.72E-01	5.88E-01	6.13E-01	4.99E-02	5.07E-01	4.85E-01	1.48E-01
			4.89E-01			4.17E-01			3.49E-01			4.60E-01			4.12E-01	
	**Rank**		5			6			9			8			9	
C_2	**Score**	9.33E-01	4.87E-01	1.13E-01	6.76E-01	4.83E-01	1.28E-01	2.69E-01	4.70E-01	3.93E-01	4.86E-01	4.67E-01	4.62E-01	1.22E-01	4.11E-01	1.76E-01
			6.17E-01			4.91E-01			3.50E-01			4.75E-01			2.08E-01	
	**Rank**		11			11			11			10			3	
C_3	**Score**	9.33E-01	6.15E-01	1.88E-01	5.08E-01	5.54E-01	2.46E-01	2.29E-01	4.89E-01	1.60E-01	5.37E-01	4.81E-01	5.21E-01	5.75E-01	3.69E-01	2.63E-01
			6.67E-01			4.54E-01			2.77E-01			5.19E-01			4.46E-01	
	**Rank**		13			7			1			14			10	
C_4	**Score**	4.77E-01	5.68E-01	1.72E-01	5.38E-01	5.20E-01	2.40E-01	2.38E-01	5.36E-01	3.89E-01	4.99E-01	5.15E-01	5.56E-01	4.07E-01	5.75E-01	4.25E-01
			4.23E-01			4.59E-01			3.50E-01			5.17E-01			4.54E-01	
	**Rank**		2			9			10			13			11	
C_5	**Score**	9.67E-01	5.16E-01	2.90E-01	5.31E-01	3.89E-01	5.11E-01	3.22E-01	4.84E-01	5.61E-01	3.69E-01	4.96E-01	4.22E-01	2.82E-01	5.40E-01	2.77E-01
			6.85E-01			4.90E-01			4.22E-01			4.14E-01			3.45E-01	
	**Rank**		14			10			15			5			6	
C_6	**Score**	9.67E-01	5.92E-01	2.39E-01	6.82E-01	4.33E-01	3.81E-01	2.78E-01	4.45E-01	2.42E-01	4.99E-01	4.54E-01	5.06E-01	5.24E-01	4.42E-01	4.46E-01
			6.91E-01			5.44E-01			3.11E-01			4.89E-01			4.84E-01	
	**Rank**		15			13			8			12			14	
C_7	**Score**	4.79E-01	5.80E-01	4.71E-01	4.02E-01	4.58E-01	2.82E-01	3.11E-01	4.26E-01	4.24E-01	4.46E-02	5.49E-01	5.66E-01	6.35E-01	4.39E-01	4.28E-01
			5.02E-01			3.86E-01			3.68E-01			3.01E-01			5.34E-01	
	**Rank**		6			2			13			2			15	
C_8	**Score**	4.33E-01	5.52E-01	1.75E-01	3.33E-01	4.01E-01	4.03E-01	2.39E-01	5.80E-01	1.65E-01	5.16E-01	3.80E-01	3.59E-01	1.69E-01	6.15E-01	1.80E-01
			3.98E-01			3.67E-01			3.05E-01			4.42E-01			2.83E-01	
	**Rank**		1			1			6			6			5	
C_9	**Score**	8.69E-01	4.59E-01	1.37E-01	5.28E-01	5.21E-01	4.67E-01	2.00E-01	5.46E-01	1.67E-01	5.85E-01	5.24E-01	4.55E-01	3.87E-02	4.85E-01	2.69E-01
			5.84E-01			5.11E-01			2.79E-01			5.37E-01			2.08E-01	
	**Rank**		8			12			3			15			4	
C_{10}	**Score**	4.74E-01	4.45E-01	4.87E-01	3.55E-01	5.11E-01	4.10E-01	2.35E-01	5.21E-01	4.61E-01	4.74E-01	5.27E-01	3.52E-01	6.04E-01	5.31E-01	1.31E-01
			4.70E-01			4.08E-01			3.63E-01			4.57E-01			4.68E-01	
	**Rank**		4			3			12			7			13	
C_{11}	**Score**	7.67E-01	4.64E-01	1.43E-01	4.04E-01	4.27E-01	4.26E-01	2.84E-01	4.86E-01	4.54E-01	5.25E-01	5.84E-01	3.03E-01	4.37E-01	4.79E-01	5.00E-01
			5.35E-01			4.15E-01			3.77E-01			4.84E-01			4.63E-01	
	**Rank**		7			4			14			11			12	
C_{12}	**Score**	9.67E-01	3.65E-01	1.61E-01	4.63E-01	5.17E-01	2.21E-01	2.52E-01	4.21E-01	1.85E-01	5.69E-01	6.05E-01	1.34E-01	6.54E-02	4.72E-01	1.94E-01
			6.15E-01			4.16E-01			2.78E-01			4.69E-01			1.99E-01	
	**Rank**		9			5			2			9			2	
C_{13}	**Score**	5.15E-01	6.03E-01	1.27E-01	6.50E-01	4.77E-01	4.05E-01	2.19E-01	4.82E-01	2.05E-01	2.81E-01	4.77E-01	2.69E-01	3.40E-02	4.59E-01	2.55E-01
			4.40E-01			5.45E-01			2.81E-01			3.27E-01			1.95E-01	
	**Rank**		3			14			4			3			1	
C_{14}	**Score**	9.67E-01	4.12E-01	1.17E-01	5.44E-01	4.74E-01	2.73E-01	1.92E-01	5.49E-01	2.10E-01	4.42E-02	4.91E-01	4.21E-01	2.67E-01	5.34E-01	4.38E-01
			6.15E-01			4.59E-01			2.86E-01			2.50E-01			3.77E-01	
	**Rank**		10			8			5			1			7	
C_{15}	**Score**	9.33E-01	4.91E-01	2.84E-01	5.84E-01	5.47E-01	5.61E-01	2.32E-01	5.04E-01	2.68E-01	3.73E-01	3.59E-01	3.98E-01	4.03E-01	5.84E-01	2.34E-01
			6.60E-01			5.69E-01			3.09E-01			3.76E-01			4.06E-01	
	**Rank**		12			15			7			4			8	

Table 4: The ranking of algorithms APO, MOA, SBOA, EOA, and TVETBO for the DDM.

		**APO**			**MOA**			**SBOA**			**EOA**			**TVETBO**		
		ν_1	ν_2	ν_3	ν_1	ν_2	ν_3	ν_1	ν_2	ν_3	ν_1	ν_2	ν_3	ν_1	ν_2	ν_3
C_1	**Score**	6.71E-01	5.03E-01	2.24E-01	9.67E-01	4.66E-01	4.72E-01	5.34E-01	4.83E-01	5.46E-01	2.57E-01	4.66E-01	2.54E-01	6.20E-01	6.43E-01	1.12E-01
			5.17E-01			7.18E-01			5.24E-01			3.09E-01			4.99E-01	
	**Rank**		13			12			10			11			5	
C_2	**Score**	5.13E-01	4.48E-01	3.64E-01	5.05E-01	4.60E-01	2.83E-01	5.06E-01	5.31E-01	4.68E-01	1.10E-01	4.87E-01	4.56E-01	6.96E-01	4.96E-01	4.45E-01
			4.60E-01			4.39E-01			5.03E-01			2.91E-01			5.83E-01	
	**Rank**		11			2			8			8			13	
C_3	**Score**	2.84E-01	5.55E-01	1.46E-01	9.67E-01	4.56E-01	2.79E-01	9.67E-01	6.33E-01	4.96E-01	5.09E-01	4.86E-01	4.47E-01	5.20E-01	5.28E-01	2.94E-01
			3.18E-01			6.67E-01			7.66E-01			4.88E-01			4.66E-01	
	**Rank**		5			10			15			12			2	
C_4	**Score**	5.74E-01	3.95E-01	2.42E-01	4.98E-01	5.71E-01	5.44E-01	6.39E-01	3.86E-01	5.96E-01	1.53E-01	3.59E-01	4.78E-01	4.95E-01	3.78E-01	3.49E-01
			4.46E-01			5.28E-01			5.65E-01			2.86E-01			4.29E-01	
	**Rank**		9			5			12			7			1	
C_5	**Score**	4.55E-01	6.01E-01	1.20E-01	5.44E-01	7.02E-01	3.09E-01	5.69E-01	4.62E-01	6.21E-01	1.51E-01	5.04E-01	4.23E-01	6.31E-01	5.42E-01	6.63E-01
			4.08E-01			5.25E-01			5.55E-01			3.07E-01			6.17E-01	
	**Rank**		6			4			11			10			15	
C_6	**Score**	2.12E-01	4.57E-01	2.71E-01	9.67E-01	8.12E-01	5.96E-01	4.75E-01	5.02E-01	1.81E-01	7.20E-02	4.05E-01	2.17E-01	5.91E-01	5.33E-01	3.34E-01
			2.88E-01			8.35E-01			4.09E-01			1.92E-01			5.12E-01	
	**Rank**		4			15			3			1			7	
C_7	**Score**	6.12E-01	4.66E-01	1.45E-01	4.42E-01	4.93E-01	5.39E-01	4.42E-01	4.72E-01	5.85E-01	1.33E-01	4.14E-01	3.33E-01	6.31E-01	4.13E-01	3.97E-01
			4.59E-01			4.79E-01			4.85E-01			2.53E-01			5.18E-01	
	**Rank**		10			3			7			4			8	
C_8	**Score**	5.17E-01	4.51E-01	1.92E-01	5.07E-01	7.01E-01	4.06E-01	4.44E-01	5.02E-01	9.49E-02	1.18E-01	5.98E-01	3.89E-01	6.43E-01	4.30E-01	4.21E-01
			4.19E-01			5.31E-01			3.71E-01			3.06E-01			5.35E-01	
	**Rank**		8			6			2			9			10	
C_9	**Score**	1.51E-01	3.98E-01	1.11E-01	5.13E-01	7.35E-01	6.24E-01	3.85E-01	4.55E-01	1.64E-01	6.79E-01	5.73E-01	1.97E-01	6.05E-01	5.27E-01	2.76E-01
			2.03E-01			5.96E-01			3.47E-01			5.32E-01			5.03E-01	
	**Rank**		2			7			1			14			6	
C_{10}	**Score**	5.79E-01	4.26E-01	3.84E-01	9.67E-01	4.92E-01	5.08E-01	4.94E-01	9.01E-01	8.17E-01	1.22E-01	4.68E-01	2.35E-01	7.29E-01	4.12E-01	5.26E-02
			4.92E-01			7.33E-01			6.77E-01			2.37E-01			4.81E-01	
	**Rank**		12			14			13			3			3	
C_{11}	**Score**	4.17E-01	5.31E-01	3.09E-01	6.69E-01	6.37E-01	6.20E-01	5.03E-01	4.89E-01	5.37E-01	5.03E-02	4.61E-01	2.98E-01	5.97E-01	3.17E-01	4.35E-01
			4.18E-01			6.49E-01			5.08E-01			2.15E-01			4.87E-01	
	**Rank**		7			8			9			2			4	
C_{12}	**Score**	1.16E-01	5.19E-01	3.83E-01	9.18E-01	6.55E-01	4.22E-01	4.32E-01	5.29E-01	5.20E-01	6.69E-01	4.57E-01	2.12E-01	6.77E-01	5.26E-01	3.22E-01
			2.83E-01			7.28E-01			4.78E-01			5.02E-01			5.50E-01	
	**Rank**		3			13			6			13			11	
C_{13}	**Score**	7.10E-01	5.66E-01	2.01E-01	5.27E-01	4.73E-01	2.12E-01	3.68E-01	5.17E-01	5.02E-01	8.98E-02	4.36E-01	4.36E-01	7.55E-01	3.13E-01	5.18E-01
			5.47E-01			4.35E-01			4.39E-01			2.63E-01			5.86E-01	
	**Rank**		15			1			5			6			14	
C_{14}	**Score**	5.44E-01	5.11E-01	5.68E-01	9.00E-01	5.18E-01	5.50E-01	4.27E-01	4.33E-01	4.39E-01	5.10E-02	4.19E-01	5.01E-01	5.95E-01	5.83E-01	4.42E-01
			5.42E-01			7.17E-01			4.32E-01			2.55E-01			5.54E-01	
	**Rank**		14			11			4			5			12	
C_{15}	**Score**	1.27E-01	4.20E-01	1.02E-01	8.67E-01	5.05E-01	3.93E-01	9.33E-01	5.82E-01	3.36E-01	7.51E-01	5.04E-01	3.82E-01	5.99E-01	2.89E-01	5.88E-01
			1.94E-01			6.58E-01			6.96E-01			5.97E-01			5.19E-01	
	**Rank**		1			9			14			15			9	

Table 5: The ranking of algorithms COA(1), GOA, COA(2), CPO, and GO for the TDM.

		**COA(1)**			**GOA**			**COA(2)**			**CPO**			**GO**		
		ν_1	ν_2	ν_3	ν_1	ν_2	ν_3	ν_1	ν_2	ν_3	ν_1	ν_2	ν_3	ν_1	ν_2	ν_3
C_1	**Score**	9.67E-01	6.14E-01	2.59E-01	5.74E-01	4.65E-01	1.51E-01	4.28E-01	5.15E-01	3.85E-01	5.05E-01	4.35E-01	2.25E-01	1.24E-01	4.39E-01	2.57E-01
			7.02E-01			4.41E-01			4.39E-01			4.18E-01			2.36E-01	
	**Rank**		15			3			13			10			4	
C_2	**Score**	7.36E-01	5.40E-01	1.37E-01	5.34E-01	5.84E-01	2.37E-01	2.69E-01	5.45E-01	1.56E-01	4.65E-01	4.85E-01	3.73E-01	1.49E-01	6.37E-01	1.08E-01
			5.38E-01			4.72E-01			3.10E-01			4.47E-01			2.61E-01	
	**Rank**		2			5			1			12			6	
C_3	**Score**	6.37E-01	5.45E-01	3.33E-01	4.86E-01	3.90E-01	3.01E-01	3.27E-01	5.59E-01	4.21E-01	4.63E-01	5.55E-01	3.58E-01	3.27E-01	3.31E-01	1.70E-01
			5.38E-01			4.15E-01			4.08E-01			4.60E-01			2.89E-01	
	**Rank**		3			2			7			13			9	
C_4	**Score**	8.67E-01	6.47E-01	3.08E-01	5.79E-01	4.51E-01	5.55E-01	2.42E-01	4.35E-01	5.90E-01	3.65E-01	5.87E-01	2.52E-01	1.04E-01	5.21E-01	1.84E-01
			6.72E-01			5.41E-01			3.77E-01			3.92E-01			2.28E-01	
	**Rank**		13			11			3			8			2	
C_5	**Score**	7.06E-01	4.50E-01	1.59E-01	3.84E-01	5.40E-01	1.81E-01	3.83E-01	5.03E-01	3.48E-01	3.43E-01	6.03E-01	3.37E-01	1.47E-01	4.51E-01	5.27E-01
			5.05E-01			3.72E-01			4.04E-01			4.07E-01			3.18E-01	
	**Rank**		1			1			5			9			11	
C_6	**Score**	8.00E-01	5.47E-01	2.39E-01	5.03E-01	4.43E-01	6.42E-01	4.00E-01	5.20E-01	3.46E-01	2.36E-01	5.57E-01	4.53E-01	4.69E-01	4.42E-01	5.29E-01
			5.97E-01			5.23E-01			4.16E-01			3.71E-01			4.77E-01	
	**Rank**		7			8			10			4			15	
C_7	**Score**	8.34E-01	5.66E-01	2.14E-01	5.62E-01	4.00E-01	5.61E-01	3.37E-01	4.96E-01	4.50E-01	4.21E-01	7.04E-01	3.07E-01	4.24E-02	6.54E-01	1.41E-01
			6.12E-01			5.21E-01			4.05E-01			4.63E-01			2.20E-01	
	**Rank**		10			7			6			14			1	
C_8	**Score**	8.39E-01	5.38E-01	2.42E-01	6.06E-01	5.28E-01	5.28E-01	3.74E-01	5.20E-01	5.30E-01	3.65E-01	4.37E-01	3.19E-01	7.75E-02	4.53E-01	4.84E-01
			6.14E-01			5.67E-01			4.49E-01			3.72E-01			2.73E-01	
	**Rank**		11			13			14			5			7	
C_9	**Score**	7.72E-01	5.73E-01	4.04E-01	6.32E-01	5.27E-01	3.47E-01	4.38E-01	4.60E-01	5.01E-01	2.88E-01	3.75E-01	7.31E-01	3.82E-01	5.01E-01	4.86E-01
			6.31E-01			5.34E-01			4.60E-01			4.21E-01			4.37E-01	
	**Rank**		12			10			15			11			14	
C_{10}	**Score**	7.75E-01	4.52E-01	3.86E-01	5.74E-01	4.88E-01	6.33E-01	3.78E-01	5.09E-01	3.74E-01	3.61E-01	5.39E-01	6.53E-01	2.50E-01	4.46E-01	1.71E-01
			5.97E-01			5.67E-01			4.10E-01			4.78E-01			2.79E-01	
	**Rank**		8			14			9			15			8	
C_{11}	**Score**	8.36E-01	4.82E-01	5.55E-01	5.95E-01	5.32E-01	5.48E-01	3.76E-01	4.93E-01	5.10E-01	2.59E-01	6.07E-01	4.09E-01	1.88E-01	5.30E-01	3.05E-01
			6.77E-01			5.68E-01			4.39E-01			3.84E-01			3.03E-01	
	**Rank**		14			15			12			7			10	
C_{12}	**Score**	7.72E-01	5.97E-01	1.66E-01	4.29E-01	6.07E-01	3.49E-01	2.37E-01	6.00E-01	6.37E-01	2.83E-01	5.36E-01	1.52E-01	4.86E-01	3.99E-01	2.71E-01
			5.77E-01			4.53E-01			4.28E-01			3.13E-01			4.11E-01	
	**Rank**		6			4			11			2			12	
C_{13}	**Score**	7.09E-01	6.07E-01	1.41E-01	5.70E-01	4.63E-01	4.34E-01	3.26E-01	4.47E-01	5.12E-01	2.68E-01	4.79E-01	4.99E-01	4.16E-01	4.21E-01	4.26E-01
			5.41E-01			5.09E-01			4.03E-01			3.79E-01			4.19E-01	
	**Rank**		4			6			4			6			13	
C_{14}	**Score**	8.70E-01	4.13E-01	2.60E-01	5.46E-01	5.50E-01	5.40E-01	3.30E-01	5.41E-01	4.36E-01	2.02E-01	5.35E-01	1.99E-01	4.40E-02	5.28E-01	3.33E-01
			6.03E-01			5.46E-01			4.10E-01			2.84E-01			2.37E-01	
	**Rank**		9			12			8			1			5	
C_{15}	**Score**	7.39E-01	4.25E-01	3.14E-01	5.23E-01	5.67E-01	4.95E-01	2.71E-01	4.55E-01	4.53E-01	2.80E-01	3.97E-01	4.20E-01	7.16E-02	5.45E-01	2.31E-01
			5.54E-01			5.27E-01			3.62E-01			3.44E-01			2.30E-01	
	**Rank**		5			9			2			3			3	

Table 6: The ranking of algorithms APO, MOA, SBOA, EOA, and TVETBO for the TDM.

		**APO**			**MOA**			**SBOA**			**EOA**			**TVETBO**		
		ν_1	ν_2	ν_3	ν_1	ν_2	ν_3	ν_1	ν_2	ν_3	ν_1	ν_2	ν_3	ν_1	ν_2	ν_3
C_1	**Score**	1.44E-01	5.49E-01	6.29E-02	9.45E-01	4.23E-01	1.97E-01	4.75E-01	4.68E-01	3.03E-01	5.19E-01	5.57E-01	6.75E-02	5.08E-01	4.03E-01	2.34E-01
			2.25E-01			6.28E-01			4.30E-01			4.15E-01			4.13E-01	
	**Rank**		3			6			3			3			3	
C_2	**Score**	1.18E-01	5.97E-01	5.52E-01	9.67E-01	4.67E-01	5.49E-01	4.94E-01	5.42E-01	2.28E-01	5.72E-01	6.16E-01	5.53E-01	6.19E-01	5.99E-01	2.44E-01
			3.46E-01			7.37E-01			4.40E-01			5.78E-01			5.20E-01	
	**Rank**		14			11			4			15			8	
C_3	**Score**	1.74E-01	4.82E-01	5.34E-01	9.67E-01	5.19E-01	3.67E-01	9.67E-01	1.44E-01	3.37E-01	2.97E-01	5.63E-01	4.11E-01	5.27E-01	5.14E-01	4.34E-01
			3.41E-01			7.05E-01			6.03E-01			3.92E-01			5.01E-01	
	**Rank**		13			9			12			2			7	
C_4	**Score**	8.19E-02	5.31E-01	3.65E-01	4.49E-01	5.57E-01	5.89E-01	9.00E-01	1.24E-01	3.79E-01	4.59E-01	6.14E-01	4.22E-01	5.98E-01	5.21E-01	6.66E-01
			2.65E-01			5.11E-01			5.76E-01			4.88E-01			5.96E-01	
	**Rank**		6			2			11			12			13	
C_5	**Score**	1.99E-01	4.41E-01	9.38E-02	9.67E-01	5.62E-01	5.83E-01	9.67E-01	8.27E-01	4.60E-01	4.65E-01	4.61E-01	3.91E-01	4.55E-01	5.04E-01	4.43E-01
			2.33E-01			7.69E-01			8.05E-01			4.46E-01			4.64E-01	
	**Rank**		4			15			14			9			5	
C_6	**Score**	6.95E-02	5.70E-01	1.39E-01	5.31E-01	7.25E-01	6.50E-01	9.00E-01	1.37E-01	2.97E-01	4.41E-01	4.77E-01	3.57E-01	5.24E-01	5.33E-01	3.89E-01
			2.12E-01			6.09E-01			5.59E-01			4.29E-01			4.92E-01	
	**Rank**		2			4			10			5			6	
C_7	**Score**	2.58E-01	3.94E-01	8.41E-02	9.67E-01	5.38E-01	5.82E-01	4.85E-01	5.14E-01	5.08E-01	5.25E-01	5.88E-01	5.73E-01	6.50E-01	4.83E-01	5.08E-01
			2.48E-01			7.63E-01			4.98E-01			5.53E-01			5.73E-01	
	**Rank**		5			14			8			14			12	
C_8	**Score**	4.43E-02	5.75E-01	1.24E-01	9.44E-01	6.06E-01	3.71E-01	5.75E-01	5.81E-01	2.60E-01	4.72E-01	3.56E-01	2.37E-01	4.86E-01	4.67E-01	6.60E-01
			1.97E-01			7.16E-01			4.98E-01			3.84E-01			5.25E-01	
	**Rank**		1			10			7			1			10	
C_9	**Score**	1.39E-01	4.79E-01	3.56E-01	3.90E-01	5.79E-01	5.45E-01	5.40E-01	4.90E-01	3.28E-01	4.90E-01	5.57E-01	1.94E-01	5.74E-01	5.25E-01	5.07E-01
			2.79E-01			4.76E-01			4.75E-01			4.33E-01			5.45E-01	
	**Rank**		8			1			5			6			11	
C_{10}	**Score**	1.64E-01	5.06E-01	4.21E-01	9.67E-01	5.56E-01	5.31E-01	5.44E-01	4.97E-01	3.83E-01	4.97E-01	4.63E-01	4.77E-01	4.71E-01	4.67E-01	1.34E-01
			3.14E-01			7.55E-01			4.92E-01			4.83E-01			3.86E-01	
	**Rank**		10			13			6			11			1	
C_{11}	**Score**	2.20E-01	5.99E-01	5.78E-01	9.67E-01	5.90E-01	4.77E-01	9.67E-01	8.83E-01	4.76E-01	4.89E-01	3.97E-01	3.59E-01	6.64E-01	5.70E-01	5.00E-01
			4.04E-01			7.50E-01			8.23E-01			4.33E-01			6.00E-01	
	**Rank**		15			12			15			7			14	
C_{12}	**Score**	1.52E-01	4.12E-01	3.84E-01	9.11E-01	6.08E-01	9.26E-02	5.25E-01	6.92E-01	3.68E-01	4.46E-01	5.70E-01	2.87E-01	7.63E-01	5.30E-01	4.70E-01
			2.75E-01			6.31E-01			5.27E-01			4.37E-01			6.31E-01	
	**Rank**		7			7			9			8			15	
C_{13}	**Score**	1.79E-01	4.45E-01	5.48E-01	9.67E-01	3.84E-01	1.22E-01	4.56E-01	4.32E-01	2.21E-01	4.69E-01	5.92E-01	1.79E-01	5.01E-01	5.04E-01	3.44E-01
			3.38E-01			6.10E-01			3.91E-01			4.27E-01			4.63E-01	
	**Rank**		12			5			2			4			4	
C_{14}	**Score**	1.98E-01	5.18E-01	4.06E-01	9.23E-01	4.51E-01	1.02E-01	9.33E-01	9.22E-01	1.80E-01	4.05E-01	4.10E-01	6.24E-01	5.20E-01	4.70E-01	4.68E-02
			3.30E-01			6.00E-01			7.42E-01			4.61E-01			3.89E-01	
	**Rank**		11			3			13			10			2	
C_{15}	**Score**	1.59E-01	4.25E-01	4.79E-01	9.33E-01	5.65E-01	1.37E-01	3.28E-01	2.67E-01	1.77E-01	5.42E-01	5.60E-01	4.13E-01	6.06E-01	4.83E-01	3.97E-01
			3.05E-01			6.42E-01			2.75E-01			5.14E-01			5.23E-01	
	**Rank**		9			8			1			13			9	

Table 7: The ranking of algorithms COA(1), GOA, COA(2), CPO, and GO for the PVMM.

		COA(1)			GOA			COA(2)			CPO			GO		
		ν_1	ν_2	ν_3	ν_1	ν_2	ν_3	ν_1	ν_2	ν_3	ν_1	ν_2	ν_3	ν_1	ν_2	ν_3
C_1	**Score**	1.71E-01	5.69E-01	2.23E-01	3.54E-01	4.17E-01	1.69E-01	4.81E-01	5.77E-01	2.87E-01	4.46E-02	5.23E-01	2.84E-01	3.33E-02	3.36E-01	3.87E-01
			2.83E-01			3.23E-01			4.56E-01			2.24E-01			1.97E-01	
	**Rank**		1			3			13			1			1	
C_2	**Score**	5.04E-01	5.61E-01	2.38E-01	3.29E-01	5.87E-01	3.77E-01	3.91E-01	5.97E-01	2.23E-01	2.08E-01	5.08E-01	4.06E-01	6.67E-02	5.46E-01	1.97E-01
			4.52E-01			4.06E-01			4.01E-01			3.32E-01			2.19E-01	
	**Rank**		5			10			7			11			3	
C_3	**Score**	3.36E-01	4.46E-01	3.27E-01	4.75E-01	4.49E-01	3.56E-01	5.77E-01	4.87E-01	2.62E-01	3.25E-01	6.11E-01	3.65E-01	6.55E-01	6.04E-01	4.33E-01
			3.62E-01			4.39E-01			4.76E-01			4.07E-01			5.87E-01	
	**Rank**		3			13			14			15			15	
C_4	**Score**	7.69E-01	6.13E-01	3.55E-01	1.75E-01	4.80E-01	3.55E-01	3.15E-01	5.13E-01	2.78E-01	2.42E-01	4.80E-01	3.82E-01	3.00E-01	5.37E-01	9.51E-02
			6.26E-01			2.96E-01			3.55E-01			3.37E-01			3.08E-01	
	**Rank**		14			2			5			12			8	
C_5	**Score**	6.68E-01	4.33E-01	4.81E-01	3.26E-01	5.15E-01	3.31E-01	2.93E-01	5.62E-01	4.34E-01	1.61E-01	3.32E-01	5.05E-01	6.67E-02	3.33E-01	4.45E-01
			5.62E-01			3.74E-01			3.95E-01			2.90E-01			2.28E-01	
	**Rank**		10			8			6			8			4	
C_6	**Score**	3.38E-01	5.61E-01	5.68E-01	4.06E-01	5.17E-01	5.48E-01	4.15E-01	3.77E-01	8.96E-02	8.16E-02	5.13E-01	3.71E-01	6.61E-01	3.54E-01	3.86E-01
			4.51E-01			4.69E-01			3.24E-01			2.62E-01			5.16E-01	
	**Rank**		4			15			2			5			13	
C_7	**Score**	6.69E-01	5.16E-01	2.42E-01	2.58E-01	5.71E-01	5.37E-01	3.30E-01	4.83E-01	5.00E-01	1.40E-01	3.27E-01	5.79E-01	1.67E-01	5.41E-01	4.35E-01
			5.24E-01			4.06E-01			4.11E-01			2.96E-01			3.27E-01	
	**Rank**		8			11			9			9			9	
C_8	**Score**	5.36E-01	4.73E-01	6.03E-01	3.40E-01	6.37E-01	3.77E-01	2.16E-01	5.03E-01	3.85E-01	8.64E-02	5.50E-01	5.31E-01	1.00E-01	5.50E-01	4.50E-01
			5.37E-01			4.24E-01			3.30E-01			3.13E-01			3.00E-01	
	**Rank**		9			12			3			10			7	
C_9	**Score**	2.73E-01	5.83E-01	2.42E-01	4.37E-01	5.10E-01	3.82E-01	3.59E-01	6.06E-01	4.23E-01	6.19E-02	4.40E-01	4.66E-01	1.67E-01	5.27E-01	2.51E-01
			3.42E-01			4.41E-01			4.37E-01			2.57E-01			2.78E-01	
	**Rank**		2			14			11			4			6	
C_{10}	**Score**	8.02E-01	5.75E-01	2.06E-01	2.58E-01	6.27E-01	4.35E-01	3.33E-01	5.17E-01	4.21E-01	3.64E-01	5.71E-01	2.93E-01	3.34E-01	3.97E-01	5.00E-01
			5.96E-01			3.95E-01			4.01E-01			3.98E-01			3.91E-01	
	**Rank**		11			9			8			14			11	
C_{11}	**Score**	5.34E-01	4.02E-01	5.50E-01	2.64E-01	5.51E-01	2.92E-01	4.23E-01	5.19E-01	3.81E-01	8.68E-02	4.17E-01	3.34E-01	2.00E-01	5.86E-01	5.63E-01
			5.05E-01			3.43E-01			4.37E-01			2.31E-01			3.87E-01	
	**Rank**		7			5			12			2			10	
C_{12}	**Score**	4.05E-01	5.87E-01	4.29E-01	4.07E-01	5.47E-01	1.08E-01	2.76E-01	4.52E-01	1.53E-01	8.07E-02	5.37E-01	3.78E-01	5.75E-01	4.23E-01	3.42E-01
			4.56E-01			3.67E-01			2.89E-01			2.69E-01			4.79E-01	
	**Rank**		6			7			1			7			12	
C_{13}	**Score**	7.68E-01	5.88E-01	5.01E-01	2.32E-01	5.65E-01	2.97E-01	4.46E-01	5.51E-01	4.95E-01	3.41E-02	5.01E-01	4.90E-01	1.67E-01	4.83E-01	1.48E-01
			6.56E-01			3.32E-01			4.84E-01			2.65E-01			2.41E-01	
	**Rank**		15			4			15			6			5	
C_{14}	**Score**	7.04E-01	5.40E-01	4.63E-01	3.29E-01	5.31E-01	2.74E-01	3.33E-01	5.20E-01	5.57E-01	6.12E-02	4.16E-01	4.37E-01	3.33E-02	6.13E-01	1.95E-01
			6.03E-01			3.66E-01			4.36E-01			2.44E-01			2.19E-01	
	**Rank**		12			6			10			3			2	
C_{15}	**Score**	6.36E-01	4.92E-01	6.76E-01	1.73E-01	4.47E-01	1.63E-01	4.02E-01	4.42E-01	1.38E-01	3.41E-01	5.72E-01	3.32E-01	6.86E-01	5.09E-01	3.93E-01
			6.10E-01			2.39E-01			3.46E-01			3.97E-01			5.68E-01	
	**Rank**		13			1			4			13			14	

Table 8: The ranking of algorithms APO, MOA, SBOA, EOA, and TVETBO for the PVMM.

		APO			MOA			SBOA			EOA			TVETBO		
		ν_1	ν_2	ν_3	ν_1	ν_2	ν_3	ν_1	ν_2	ν_3	ν_1	ν_2	ν_3	ν_1	ν_2	ν_3
C_1	**Score**	6.36E-02	5.31E-01	1.19E-01	3.81E-02	8.93E-01	1.62E-01	4.67E-01	1.06E-01	2.63E-01	4.68E-01	3.91E-01	6.72E-02	5.50E-01	4.78E-01	4.64E-01
			1.94E-01			2.83E-01			3.26E-01			3.48E-01			5.10E-01	
	**Rank**		2			1			1			2			13	
C_2	**Score**	5.66E-02	3.56E-01	4.16E-01	3.14E-01	7.54E-01	2.37E-01	7.34E-01	6.65E-01	4.95E-01	2.85E-01	5.10E-01	3.21E-01	4.99E-01	5.45E-01	5.37E-01
			2.21E-01			4.05E-01			6.57E-01			3.50E-01			5.20E-01	
	**Rank**		3			9			11			3			14	
C_3	**Score**	3.26E-01	3.86E-01	2.07E-01	8.57E-02	8.94E-01	4.39E-01	4.67E-01	5.54E-01	5.66E-01	6.14E-01	4.48E-01	2.66E-01	4.03E-01	4.67E-01	6.37E-02
			3.11E-01			3.76E-01			5.13E-01			4.85E-01			3.34E-01	
	**Rank**		8			7			5			11			1	
C_4	**Score**	5.08E-02	4.20E-01	2.46E-01	2.02E-01	8.46E-01	2.07E-01	8.67E-01	8.61E-01	4.19E-01	2.53E-01	5.26E-01	4.08E-01	5.07E-01	4.33E-01	5.28E-01
			1.92E-01			3.64E-01			7.53E-01			3.60E-01			4.94E-01	
	**Rank**		1			5			15			4			11	
C_5	**Score**	2.47E-01	4.30E-01	3.71E-01	6.72E-02	8.86E-01	3.58E-01	7.33E-01	7.41E-01	4.52E-01	4.66E-01	4.23E-01	2.82E-01	4.37E-01	4.80E-01	5.17E-01
			3.24E-01			3.45E-01			6.65E-01			4.10E-01			4.68E-01	
	**Rank**		9			4			12			6			8	
C_6	**Score**	3.14E-01	5.65E-01	4.76E-01	3.34E-02	9.23E-01	4.88E-01	3.33E-01	4.69E-01	4.82E-01	4.36E-01	5.64E-01	2.52E-01	5.31E-01	4.20E-01	1.79E-01
			4.17E-01			3.69E-01			4.05E-01			4.22E-01			4.15E-01	
	**Rank**		14			6			3			7			4	
C_7	**Score**	8.31E-02	4.43E-01	4.12E-01	1.32E-01	6.77E-01	4.25E-01	9.33E-01	5.47E-01	3.62E-01	4.61E-01	5.28E-01	3.75E-01	4.22E-01	5.05E-01	1.35E-01
			2.55E-01			3.41E-01			6.94E-01			4.56E-01			3.71E-01	
	**Rank**		4			3			13			10			3	
C_8	**Score**	3.82E-01	4.65E-01	2.85E-01	6.09E-01	7.14E-01	3.25E-01	5.67E-01	8.99E-01	7.54E-01	6.28E-01	4.77E-01	4.88E-01	4.44E-01	5.97E-01	3.25E-01
			3.79E-01			5.64E-01			6.97E-01			5.55E-01			4.53E-01	
	**Rank**		13			13			14			14			6	
C_9	**Score**	1.60E-01	5.37E-01	2.85E-01	3.91E-01	8.72E-01	2.12E-01	5.00E-01	2.41E-01	3.33E-01	2.36E-01	6.10E-01	9.37E-02	4.70E-01	4.29E-01	1.02E-01
			2.86E-01			4.67E-01			3.93E-01			2.94E-01			3.68E-01	
	**Rank**		7			10			2			1			2	
C_{10}	**Score**	1.95E-01	5.20E-01	5.73E-01	3.33E-02	8.53E-01	6.09E-01	7.33E-01	6.38E-01	2.45E-01	3.66E-01	5.21E-01	4.46E-01	5.72E-01	4.15E-01	3.97E-01
			3.71E-01			3.82E-01			5.87E-01			4.25E-01			4.89E-01	
	**Rank**		12			8			7			9			9	
C_{11}	**Score**	3.62E-01	6.18E-01	3.75E-01	5.51E-01	8.72E-01	1.41E-01	5.00E-01	4.14E-01	3.34E-01	3.81E-01	4.83E-01	3.42E-01	5.02E-01	5.89E-01	3.80E-01
			4.29E-01			5.29E-01			4.37E-01			3.96E-01			4.93E-01	
	**Rank**		15			11			4			5			10	
C_{12}	**Score**	2.12E-01	5.09E-01	5.01E-01	5.72E-01	9.32E-01	3.23E-01	8.34E-01	8.33E-01	6.63E-02	6.61E-01	5.62E-01	1.92E-01	4.53E-01	4.83E-01	4.67E-01
			3.58E-01			6.00E-01			6.42E-01			5.19E-01			4.64E-01	
	**Rank**		11			15			10			12			7	
C_{13}	**Score**	2.71E-01	3.67E-01	2.11E-01	3.33E-02	7.61E-01	5.33E-01	7.67E-01	5.88E-01	2.72E-01	4.66E-01	4.72E-01	2.85E-01	5.45E-01	5.13E-01	3.75E-01
			2.80E-01			3.40E-01			5.99E-01			4.22E-01			4.94E-01	
	**Rank**		6			2			8			8			12	
C_{14}	**Score**	2.56E-01	5.08E-01	3.10E-01	4.10E-01	8.44E-01	5.94E-01	6.00E-01	4.73E-01	5.20E-01	5.95E-01	4.82E-01	4.81E-01	4.33E-01	6.55E-01	1.95E-01
			3.32E-01			5.65E-01			5.48E-01			5.39E-01			4.29E-01	
	**Rank**		10			14			6			13			5	
C_{15}	**Score**	3.35E-02	4.88E-01	4.75E-01	6.56E-01	7.28E-01	1.85E-01	5.00E-01	8.66E-01	6.61E-01	6.66E-01	6.00E-01	4.44E-01	6.06E-01	5.59E-01	4.74E-01
			2.57E-01			5.56E-01			6.32E-01			5.94E-01			5.61E-01	
	**Rank**		5			12			9			15			15	
